# Contribution of independent and pleiotropic genetic effects in the metabolic syndrome in a hypertensive rat

**DOI:** 10.1371/journal.pone.0182650

**Published:** 2017-08-08

**Authors:** Man Chun John Ma, Janette M. Pettus, Jessica A. Jakoubek, Matthew G. Traxler, Karen C. Clark, Amanda K. Mennie, Anne E. Kwitek

**Affiliations:** 1 Department of Pharmacology, University of Iowa, Iowa City, Iowa, United States of America; 2 Iowa Institute of Human Genetics, University of Iowa, Iowa City, Iowa, United States of America; University of Texas Health Science Center at San Antonio, UNITED STATES

## Abstract

Hypertension is a major risk factor for cardiovascular disease, Type 2 diabetes, and end organ failure, and is often found concomitant with disorders characteristic of the Metabolic Syndrome (MetS), including obesity, dyslipidemia, and insulin resistance. While the associated features often occur together, the pathway(s) or mechanism(s) linking hypertension in MetS are not well understood. Previous work determined that genetic variation on rat chromosome 17 (RNO17) contributes to several MetS-defining traits (including hypertension, obesity, and dyslipidemia) in the Lyon Hypertensive (LH) rat, a genetically determined MetS model. We hypothesized that at least some of the traits on RNO17 are controlled by a single gene with pleiotropic effects. To address this hypothesis, consomic and congenic strains were developed, whereby a defined fragment of RNO17 from the LH rat was substituted with the control Lyon Normotensive (LN) rat, and MetS phenotypes were measured in the resultant progeny. Compared to LH rats, LH-17^LN^ consomic rats have significantly reduced body weight, blood pressure, and lipid profiles. A congenic strain (LH-17^LN^c), with a substituted fragment at the distal end of RNO17 (17q12.3; 74–97 Mb; rn4 assembly), showed differences from the LH rat in blood pressure and serum total cholesterol and triglycerides. Interestingly, there was no difference in body weight between the LH-17^LN^c and the parental LH rat. These data indicate that blood pressure and serum lipids are regulated by a gene(s) in the distal congenic interval, and could be due to pleiotropy. The data also indicate that body weight is not determined by the same gene(s) at this locus. Interestingly, only two small haplotypes spanning a total of approximately 0.5 Mb differ between the LH and LN genomes in the congenic interval. Genes in these haplotypes are strong candidate genes for causing dyslipidemia in the LH rat. Overall, MetS, even in a simplified genetic model such as the LH-17^LN^ rat, is likely due to both independent and pleiotropic gene effects.

## Introduction

Hypertension is a major risk factor for morbidity and mortality from heart disease, stroke, and renal failure. An estimated 78 million Americans are hypertensive, associated with over $45 billion in health care costs [1, 2]. Blood pressure (BP) is a complex trait, controlled by a series of networks, involving multiple organ systems, and is affected by genes and environment stimuli. Furthermore, hypertension has a heterogeneous etiology, meaning the mechanism leading to hypertension can vary greatly between patients. While genetic susceptibility is established in hypertension [3], and significant insight has been established by studying rare monogenic forms of hypertension [4], the heterogeneity of mechanisms leading to hypertension presents a challenge for identifying variants with major effects and creates a key gap in knowledge for fully understanding and effectively treating the disease.

Hypertension frequently co-exists with a series of other complex disorders including central obesity, dyslipidemia, and insulin resistance; the collection of hypertension with these other disorders is referred to as Metabolic Syndrome (MetS). Each defining feature of MetS also has a genetic component, with strong influences by environmental stimuli [5, 6]. Some of the underlying genetic factors for MetS-associated hypertension may be due to pleiotropy (single gene/variant causes multiple phenotypes). Identifying gene pleiotropy in hypertension and MetS traits is an active field, promoted by the results of genome-wide association, transcriptome, and other related studies [7–9]. However, to date the number of genes identified with pleiotropy involving blood pressure is limited, possibly due to the relatively low effect sizes of the variants identified in hypertension GWAS as well as high heterogeneity between populations.

Identification of the genetic contribution to complex disease is aided by comprehensive studies involving common and rare genetic variation, transcriptional regulation, systems biology, and model systems [10]. The Lyon Hypertensive (LH) rat is an inbred selection model of spontaneous hypertension that also exhibits phenotypes associated with MetS (high body weight, high plasma lipids, and altered insulin:glucose ratio) [11–13]. The Lyon normotensive (LN) control strain, selectively bred for normal blood pressure from the same Sprague Dawley (SD) founder colony, is genetically similar to the LH, but is normotensive, salt-resistant, and lacks any features of MetS [14, 15]. Increased body weight, left ventricular hypertrophy, and dyslipidemia are observed in LH rats [14, 16]. Further investigation determined that LH has significant increases compared to LN in plasma cholesterol, phospholipids and total triglycerides bound with HDL, LDL and VLDL, together with increased insulin and insulin/glucose ratio [12, 14, 15]. These phenotype patterns establish the LH as a MetS-susceptible rat strain.

To study the genetic components of MetS and in the LH, genome-wide linkage scans were performed in F2 intercrosses between the LH and LN strains to map multiple phenotypes, including blood pressure, pulse pressure, heart rate, body weight, relative organ weights, serum lipids, glucose, and insulin, among others [17–19]. Interestingly, rat chromosome (RNO) 17 was found to contain QTL for *multiple* MetS traits including blood pressure, body weight, and plasma measures of lipid, leptin, and insulin levels [18, 19]. These studies implicate a single chromosome is sufficient to cause MetS in LH rats. To study the independent role of RNO17 in MetS, a consomic LH-17^BN^ strain was developed, where only RNO17 from the LH rat was substituted by that of the sequenced BN rat (BN/NHsdMcwi) [20]. This strain was found to have reduced blood pressure, body weight, and plasma triglycerides, confirming the independent role of LH RNO17 in MetS. However, not all phenotypes were recapitulated when substituting RNO17 with the BN allele. The BN genome is genetically distinct from both LH and LN strains used for the mapping studies which make integrating with previous genetic studies a challenge, and a whole chromosome substitution is not ideal for gene discovery or to study the role of pleiotropy in LH MetS. Therefore, a consomic LH-17^LN^ strain was developed and studied for traits defining MetS. This strain was then used to generate congenic substrains that could address whether independent or pleiotropic gene effects underlie the MetS traits on LH RNO17.

## Results

### Phenotyping consomic and congenic rats identifies a locus regulating blood pressure and lipids

**Fig 1:**
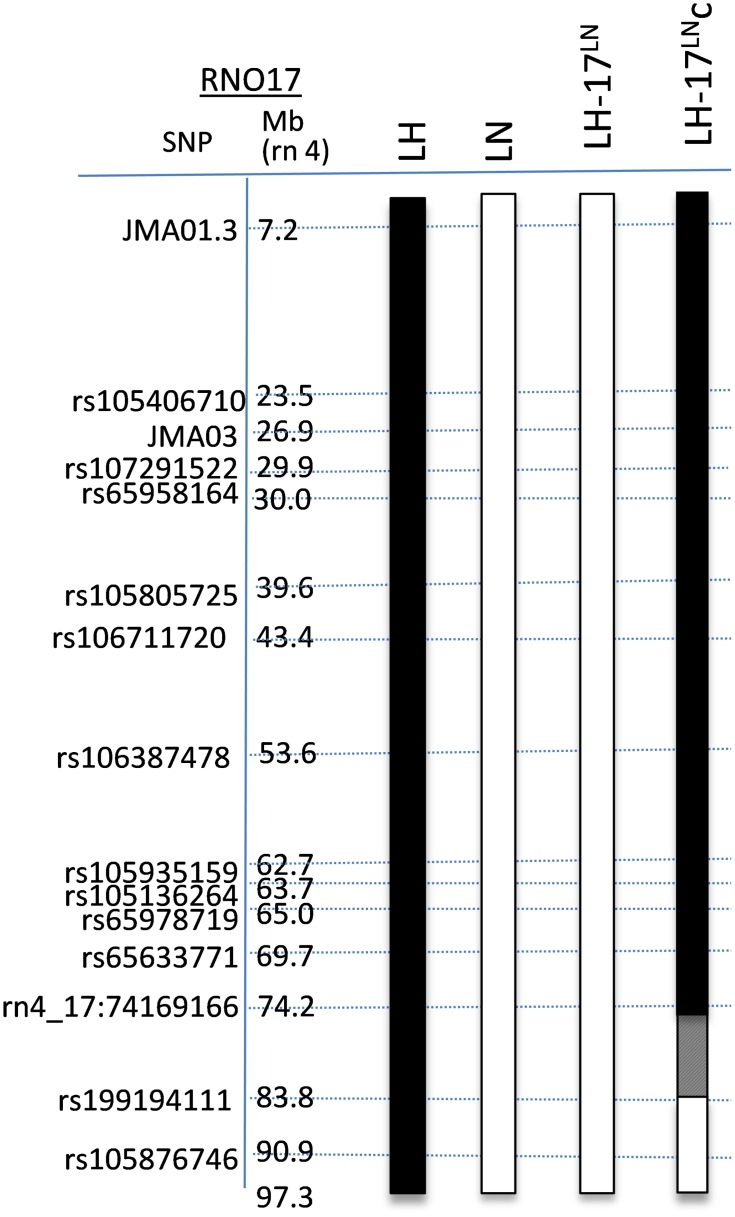
Genetic make-up of RNO17 in consomic and congenic LH-17^LN^ rats derived from LH and LN rats. Single nucleotide polymorphisms (SNPs) used for marker-selected breeding on the left alongside genome position (Mb; rn4). The RNO17 genotypes for each strain are depicted in the bars, colored according to their genome content from the parent of origin. Strain name is at the top of each bar. Note that all chromosomes except RNO17 are fixed for the LH genome. Black box = LH genome; White box = LN genome. Hatched region indicates the recombination breakpoint.

Previous studies implicated rat chromosome 17 (RNO17) in the genetic regulation of several features of MetS in the LH rat. However, F2 offspring from the mapping studies could not readily lead to gene discovery, or discern if the phenotypes mapping to RNO17 were a result of gene pleiotropy or independent gene effects. To generate a model system for gene discovery and functional studies, the ‘normal’ LN RNO17, or a fragment of LN RNO17, was transferred onto the background genome of the LH rat by marker-assisted selective breeding [21]. Fig 1 details the map of RNO17 in the consomic LH-Chr17^LN^/Aek (LH-17^LN^) and congenic LH.LH-Chr 17^LN^-(rs199194111-rs105876746)/Aek (LH-17^LN^c) strains. The consomic strain has all of RNO17 derived from the LN rat, while the rest of the genome, including the sex chromosomes and mitochondrial DNA, are from the LH rat. The LH-17^LN^c congenic carries the LH allele of RNO17 from the proximal end of the chromosome to 74.2 Mb and the LN allele from 83.8 Mb to the distal end of the chromosome. The hatched region between 74.2 to 83.8 Mb contains the recombination breakpoint.

Phenotypes including blood pressure and heart rate, serum lipid levels, and body weights and growth were determined in consomic, congenic and LH control rats.

#### Blood pressures

**Fig 2:**
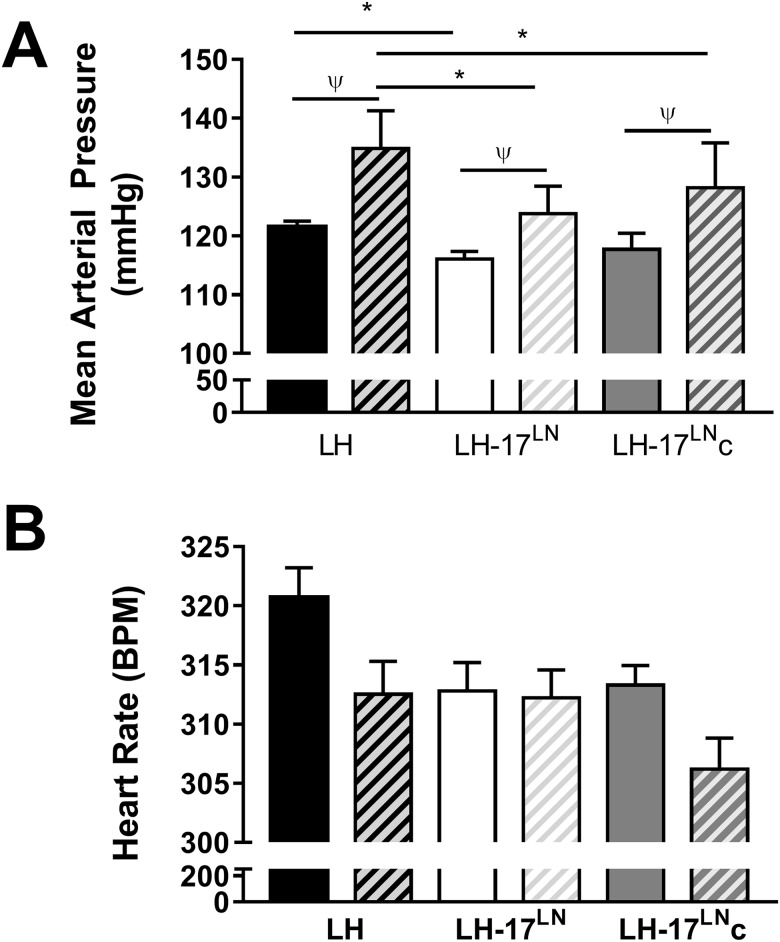
Blood pressure is affected by substitution with LN genome regions of RNO17 in male LH rats on chow and high salt diets. Mean arterial pressure (A) and heart rate (B) in LH parental, LH-17^LN^ consomic, and LH-17^LN^c congenic rats on a normal chow diet (solid bars) or after two weeks of a 4% NaCl diet (hatched bars. MAP = mean arterial pressure; HR = heart rate. LH: n = 20; LH-17^LN^: n = 9; LH-17^LN^c: n = 6. Data displayed as mean ± SE. Two-way ANOVA indicated significant effects of both diet and strain, with no significance for diet x strain interaction. ANOVA was followed by post-hoc Holms-Sidak multiple comparison tests. ^ψ^ p < 0.05 chow vs 4% salt diet; * p < 0.05 vs LH rat.

Blood pressure (including Mean Arterial Pressure (MAP), Systolic Blood Pressure (SBP), and Diastolic Blood pressure (DBP)) and heart rate (HR) were measured for 72 hours before and after two weeks on a 4% salt diet. Both 3-day and hourly averages were determined in each rat to determine heart rate and blood pressures before and after a salt diet. Prior to a high salt diet, MAP and SBP in LH-17^LN^ rats is reduced compared to LH rats (Fig 2A, S1 Fig). After 2 weeks on 4% salt, LH-17^LN^ rats have reduced MAP, SBP, and DBP compared to the LH strain (Fig 2A, S1 Fig). Both genotype and diet affects the MAP in the consomic, indicating the substitution of LH RNO17 with the LN genome influences both basal blood pressure and salt-sensitivity. Heart rate in the consomic did not significantly differ on either diet, although there was a trend for a lower decrease in heart rate after high dietary salt in LH-17^LN^ compared to the LH rats (Fig 2B).

When fed chow, neither MAP nor HR differs in the congenic LH-17^LN^c rats compared to the LH control strain (Fig 2A, S1 Fig). However, significant differences in blood pressure were found between the LH-17^LN^c and LH rats after a high salt diet(Fig 2A, S1 Fig), possibly due to salt resistance from the LN allele in the congenic region. Interestingly, the pressure differences between the congenic LH-17^LN^c and LH rats are more pronounced during the light period (6 am– 6 pm), when the rats are inactive (S1 Fig). The LH rat has a ‘non-dipping’ blood pressure pattern that can be a risk factor for cardiovascular events, which is resolved in the congenic LH-17^LN^c strain. The blood pressure changes are independent of heart rate, as there are not significant differences between the congenic and LH strains (Fig 2B).

#### Blood glucose, triglyceride, and cholesterol levels

**Fig 3:**
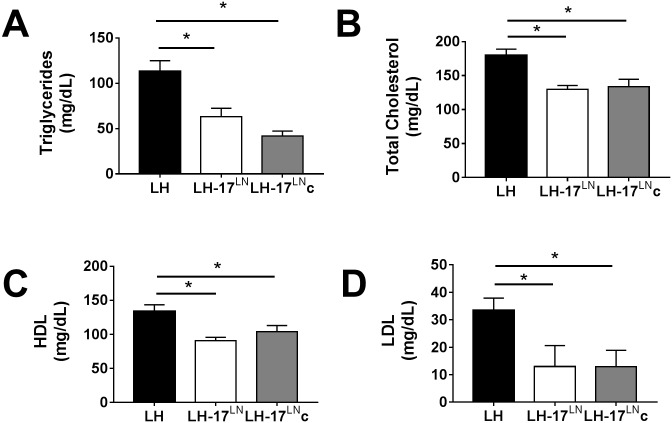
Serum triglyceride and cholesterol levels are affected by substitution of LN genome regions of RNO17 in male LH rats. The substitution of all of RNO17 (LH-17^LN^ consomic) or 17q12.3 (LH-17^LN^c congenic) reduces serum triglyceride levels (3A; LH: n = 20; LH-17^LN^: n = 6; LH-17^LN^c: n = 6), Serum total cholesterol (B), HDL (C), and LDL (D) cholesterol levels were also decreased compared to the LH rat. LH: n = 7; LH-17^LN^: n = 5; LH-17^LN^c: n = 5. Data displayed as mean ± SE. *p < 0.05 by one-way ANOVA and Holms-Sidak multiple comparison test vs LH.

Blood glucose and serum cholesterol—total, high-density lipoprotein (HDL), and low-density lipoprotein (LDL)—and triglyceride levels were determined in LH-17^LN^, LH-17^LN^c, and LH rats. Blood glucose (mg/dL) did not differ between the groups (LH: 88 ± 3.4; LH-17^LN^: 98 ± 15.3; LH-17^LN^c: 83 ± 6.4). Compared to LH rats, LH-17^LN^ rats have reduced total cholesterol, HDL, LDL, and triglyceride levels (Fig 3A–3D). Interestingly, substituting only the distal end of RNO17 was also sufficient to reduce serum cholesterol (total, HDL and LDL) and triglyceride levels (Fig 3A–3D), indicating a strong genetic factor in distal RNO17 regulating serum lipid levels. Both HDL and LDL are downregulated in the consomic and congenic strains compared to LH rats. Although there is a ~30% decrease in HDL in the consomic and congenic strains compared to the LH rat (LH: 134 ± 9.5 mg/dL; LH-17^LN^: 90.6 ± 5.1 mg/dL; LH-17^LN^c: 103.8 ± 9.2 mg/dL) the decrease in LDL is more pronounced with a >60% reduction in both the LH-17^LN^ and LH-17^LN^c strains compared to the LH rat (LH: 33.5 ± 4.4 mg/dL; LH-17^LN^: 12.6 ± 7.8 mg/dL; LH-17^LN^c: 12.7 ± 6.1 mg/dL.

#### Body measures

**Fig 4:**
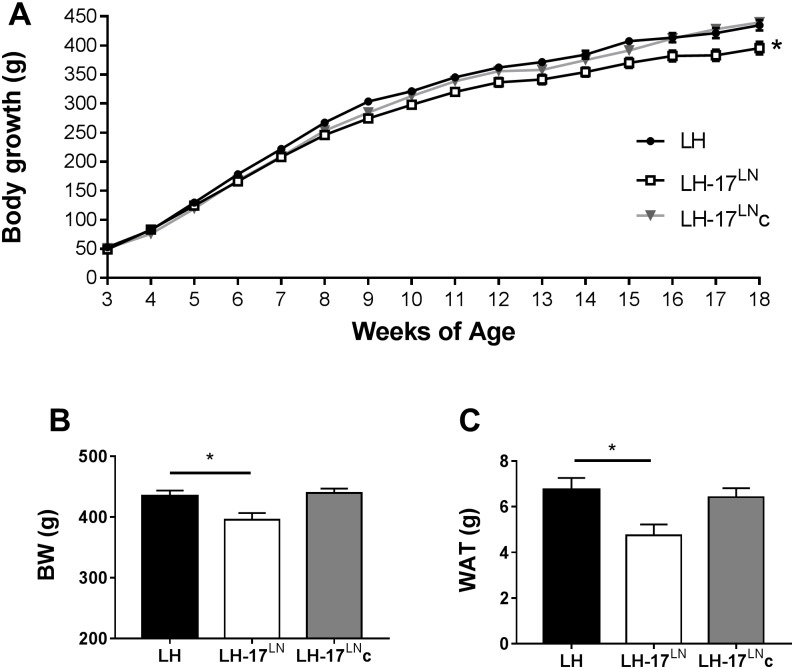
Body weight and adiposity are reduced in male LH-17^LN^ consomic but not in male LH-17^LN^c congenic rats. A. Growth curves from 3–18 weeks of age. B. Body weight at 18 weeks of age. C. Mass of abdominal white adipose tissue (perirenal + gonadal fat pads) at 18 weeks of age. BW = body weight; WAT = abdominal white adipose tissue. LH: n = 10; LH-17^LN^: n = 9; LH-17^LN^c: n = 6. Data displayed as mean ± SE. *p < 0.05 by either two-way (Growth) or one-way (BW and WAT) ANOVA followed by Holms-Sidak multiple comparison test vs LH.

Compared to LH parental rats, the consomic LH-17^LN^ rats have significantly reduced body weight starting at 8 weeks of age and persisting throughout the protocol (Fig 4A & 4B). Unlike the LH-17^LN^ strain, the congenic LH-17^LN^c strain has no difference in body weight compared to the LH parental strain. Similarly, white adipose tissue (WAT) mass is significantly reduced only in LH-17^LN^ compared to LH rats, while the LH-17^LN^c congenic is similar to the parental LH strain (Fig 4C). These data indicate body weight and adiposity are regulated by genetic factors on RNO17, but not by the distal fragment of RNO17.

### Genome analysis on RNO17 identifies putative candidate genes for the LH-17LN c phenotype

Using data from the sequenced genomes of LH and LN rat strains, we previously determined the strains differ by <0.02% at the sequence level [22]. Moreover, by analyzing the genome distribution of sequence variants between the two strains, we determined that over 98% of the single nucleotide variants (SNVs) between the strains cluster into ancestrally divergent haplotype blocks with an average length of < 1Mb [23]. These haplotypes, from differing ancestral genomes are presumed to contain the genetic variation causing the phenotypic variation between the LH and LN strains due to selective breeding, and can assist in refining genome intervals introgressed in consomic and congenic strains to identify candidate causal genes.

**Fig 5:**
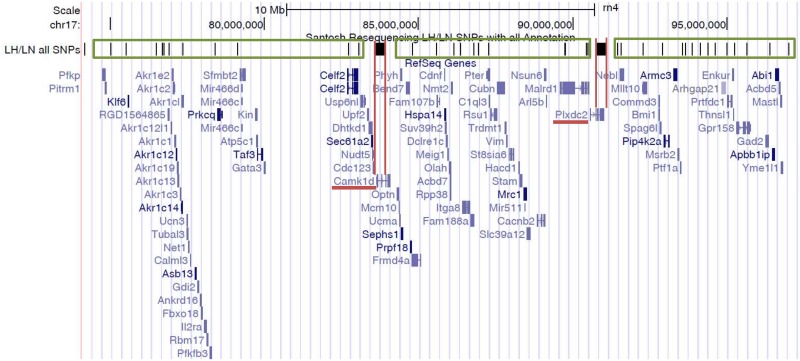
Genome region substituted in the LH-17^LN^c congenic strain reveals *Camk1d* and *Plxdc2* as positional candidate genes. Screen shot from the UCSC Browser (http://genome.ucsc.edu) of the genome interval on 17q12.3 (rn4) substituted in the LH-17^LN^c congenic strain. Scale is at the top. LH/LN all SNPs track shows SNVs as black ticks. RefSeq genes are the bottom track. Green boxes denote regions of shared ancestry between LH and LN genomes with low sequence diversity. Red bars mark small genome regions of high SNV density that are of different ancestral origin in LH and LN genomes. The only genes in these ancestrally different regions—*Camk1d* and *Plxdc2*—are underlined in red.

The interval of RNO17 that differs between the LH-17^LN^c congenic and the LH parental strain includes the distal ~13 Mb of the chromosome, with the proximal recombination breakpoint between 74.2 and 83.8 Mb (rn4). To narrow the region containing the causal gene(s) at this locus (74–97 Mb on rn4 assembly), SNVs in the introgressed interval were evaluated using the whole genome sequence of the LH and LN strains (Fig 5; S2 Table). There are 1117 SNVs between the LH and LN genome sequence in the congenic interval (rn4). However, 1079 (96.6%) of those variants are tightly clustered within 2 small blocks spanning 0.33 and 0.26 Mb, which are surrounded by 3 large intervals of DNA spanning 9.42, 6.89, and 6.25 Mb having shared ancestry and thus almost no genetic variation.

The small genome blocks define haplotypes that are ancestrally different between LH and LN and are most likely to have functional genetic variation affecting the phenotype in the LH-17^LN^c congenic due to the selective breeding for blood pressure in the parental strains. The two divergent haplotypes each contain only a single gene. The first haplotype that differs between the LH and LN genomes has 515 variants in a 0.33 Mb region (83.58–83.91 Mb), and contains the proximal 2 exons and 40 kb upstream of *Camk1d*. While no variants were identified in the coding regions of *Camk1d*, there were several variants falling within evolutionarily conserved sites that could be involved in gene regulation. The second divergent haplotype has 564 variants in a 0.26 Mb region (90.78–91.04 Mb), containing the distal part of *Plxdc2* (exons 3–14) and 25 kb downstream. As with *Camk1d*, no coding variants were identified in *Plxdc2* but many potential regulatory variants exist.

The three genome regions of *shared* ancestry in the LH-17^LN^c congenic interval collectively have only 38 SNVs between the LH and LN: The first region spans 9.42 Mb and has 11 SNVs between the LH and LN genomes; the second region spans 6.89 Mb and also has 11 SNVs; the third region spans 6.25 Mb and contains 16 variants. These 38 SNVs spanning over 22 Mb are likely to be variants that arose subsequent to the selection and inbreeding of the strains. Most of these SNVs are intergenic, although ten variants fall within gene regions: *RGD1564865*, *Akr1cl*, *Celf2*, *Pter*, *Nebl*, *Mllt10*, and *Gpr15*8 each have a single intronic variant; *Malrd1* has two intronic variants; and *Frmd4a* has a single synonymous variant. There are no variants that would be expected to change amino acid sequence of the genes and there is no evidence of structural variation between the strains in the congenic interval.

### qPCR in selected tissues

**Fig 6:**
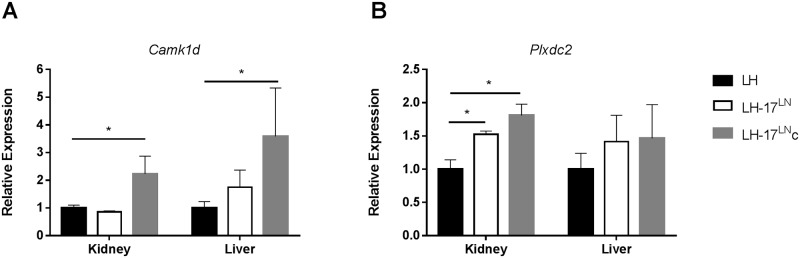
mRNA expression in kidney and liver tissue from LH-17^LN^, LH-17^LN^c,and LH strains. *Camk1d* mRNA expression differs in LH-17^LN^c vs LH strains in kidney and liver (A); expression in the consomic was not significantly different from the parental LH strain. *Plxdc2* mRNA is upregulated in kidney of both LH-17^LN^ and LH-17^LN^c compared to LH rats, but not in liver. LH: n = 7–10; LH-17^LN^: n = 5–6; LH-17^LN^c: n = 5–6. Data displayed as mean ± SE. *p < 0.05 by one-way ANOVA followed by Holms-Sidak multiple comparison test vs LH.

Because of the high number of SNVs in *Camk1d* and *Plexdc2*, mRNA expression analysis of *Camk1d* and *Plexdc2* in consomic, congenic, and LH rats was performed in liver and kidney tissue to determine if SNVs in the congenic interval cause cis-regulation of gene expression. The mRNA expression of *Camk1d* differed in LH-17^LN^c rats compared to LH control animals in both kidney and liver tissue, whereas the expression in the LH-17^LN^ consomic was similar to the LH parental strain (Fig 6A). The gene expression data suggests that *Camk1d* is cis-regulated but may also be affected by modifiers outside the congenic interval (ie residing in other regions of chromosome 17). *Plxdc2* mRNA expression was higher in LH-17^LN^ and LH-17^LN^c kidney compared to LH, but neither was different in liver (Fig 6B), suggesting tissue specific expression differences.

## Discussion

The primary hypothesis for these studies is that the MetS traits mapping to LH chromosome 17 are caused by a gene(s) with pleiotropic effects or by passenger loci fixed in the selection for hypertension in the LH strain. In this study, a consomic rat strain having an LN rat chromosome 17 on an LH rat genome background was generated to confirm previous QTL mapping studies and to generate an inbred animal model to be used for novel gene discovery. Subsequently, a congenic strain with the substitution of only 17q12.3 identified *Camk1d* and *Plxdc2* as candidate genes for hypertension and dyslipidemia in the LH strain. Of note, *CAMK1D* is an established candidate gene for type II diabetes in human GWAS, with additional evidence implicating it in hypertension [24–26]. Our studies indicate *Camk1d* is genetically regulated in the LH rat, making it an attractive animal model to better understand a pleiotropic role for this gene in important features of the metabolic syndrome. This study and our previous data suggest there are independent loci contributing to the blood pressure phenotype, which also show gene pleiotropy for other traits defining MetS.

These studies first show that substitution of LH chromosome 17 with LN chromosome 17 is sufficient to alter several components of MetS, including blood pressure on normal and high salt diet, serum cholesterol and triglycerides, body weight and adiposity. These data support many previous findings from an LH x LN F2 intercross where 23 quantitative trait involving body weight, blood pressure, and plasma lipids mapped to the same chromosome [18, 19]. Genetic studies in other rat strains have also identified loci for hypertension and other MetS traits on rat chromosome 17 [27–33]. Previously a consomic rat having a BN chromosome 17 on an LH genome background was generated [20]. That study showed a profile similar to that of the LH-17^LN^ consomic reported here, with decreased MAP compared to LH parental rats before and after a salt challenge, decreased body weight at 18 weeks of age, as well as decreased plasma triglycerides compared to their respective LH controls. However, in LH-17^BN^ strain, high salt diet resulted in blood pressure increases comparable to LH, indicating the consomic is equally salt sensitive as the LH strain. In contrast, the change in blood pressure before and after high salt is smaller in LH-17^LN^ compared to LH rats (ΔMAP: LH = 13.5 + 0.9 mmHg vs LH-17^LN^ = 7.7 + 0.7 mmHg; p <0.001), indicating resistance to salt due to the LN chromosome 17. Furthermore, the substitution with an LN chromosome 17 reduced serum cholesterol, which was not the case with the substitution with a BN chromosome 17. The phenotype differences between the LH-17^BN^ and LH-17^LN^ strains are likely due to the fact that the BN genome vastly differs from both the LH and the LN genome and may harbor its own susceptibility alleles for salt sensitivity and hypercholesterolemia which are masked in the BN genome but revealed on the LH genome background.

A major advantage of using the LN strain as a donor strain for the consomic and congenic strains lies in the close relatedness of the LH and LN strains (as compared to e.g. the BN strain). Despite their substantial phenotype differences, the LH and LN strains are among the most closely related of all characterized rat strains and share much of the same ancestral DNA derived from the founder Sprague Dawley rats used to generate both lines [22, 34]. While the selective breeding in the LH and LN strains enriches for variants causing their different blood pressure phenotypes, their relatedness results in fewer variants that could contribute to the phenotype differences [23].

To further dissect the genetic factors contributing to key features of MetS on chromosome 17 of the LH strain, a congenic strain (LH-17^LN^c) with only 17q12.3 from the LN strain on an otherwise LH rat genome was generated. This region is supported by previously identified QTL for blood pressure [30, 33]. The LH-17^LN^c strain has reduced blood pressure and serum lipid content, supporting gene pleiotropy at this locus. Previous genetic data in the LN x LN F2 intercross support the correlation between blood pressure and lipid levels regulated by this region of chromosome 17 [18]. F2 rats sharing a genotype for a marker on distal chromosome 17 showed a significant correlation between mean blood pressure and both cholesterol and triglycerides. However in both the congenic and in the F2 intercross, the 17q locus does not regulate body weight. In other words, the genetic regulation of body weight is due to factors elsewhere on chromosome 17.

The congenic strain also revealed that 17q12 alone significantly reduced serum triglycerides (even more than in the consomic strain), suggesting a strong independent genetic factor in this region. Mean arterial blood pressure in the congenic strain, while significantly lower than the LH strain, was higher than with the whole chromosome substitution. This was not unexpected; we previously identified an independent locus on 17p13 that regulates both blood pressure and body weight, and identified *RGD1562963* as the likely causal gene at that locus [19]. The identification of multiple loci regulating blood pressure on a single chromosome is likely due to the selective breeding for high blood pressure in the LH strain, resulting in the concomitant selection of genes contributing to hypertension. This and our previous studies also indicate that the loci regulating blood pressure selected for in the LH strain have pleiotropic effects on the additional phenotypes underlying MetS that map to LH chromosome 17.

The phenotype in the congenic LH-17^LN^c rats identified two candidate genes falling within the congenic region–*Camk1d* and *Plxdc2*. *Camk1d* has numerous SNVs between LH and LN rats, and is differentially expressed in the LH-17^LN^c compared to LH rats harboring those SNVs. These data make *Camk1d* an attractive candidate for being cis-regulated and for causing the phenotype differences in the congenic strain. *Camk1d* is member of the calcium/calmodulin-dependent protein kinase 1 gene family that plays role in granulocyte respiratory burst, phagocytosis, adhesion and migration [35]. It is also known to activate CREB-mediated transcriptional activation [36] and may play a role in regulating hepatocyte glycolysis and gluconeogenesis independent of insulin signaling [37]. Of important relevance to this project, variants in *Camk1d* have replicated association with Type II diabetes [24–26] and a recent study also found association of this gene to hypertension [38]. Therefore this gene is a strong functional candidate for our studies.

*Plxdc2* (Plexin domain containing 2) is less well characterized than *Camk1d*, but it has been described as highly expressed endothelial tumor marker involved in angiogenesis, and a transmembrane receptor for Pigment Epithelium Derived Factor [39, 40]. It has been associated with diabetic retinopathy [41], but not with hypertension or other MetS related traits. While an obvious link to hypertension and dyslipidemia is not apparent for this gene, it cannot be eliminated it as a candidate based upon function alone.

While these studies implicate only two genes in the congenic region, it is possible that the variants causing the phenotype are due to an unannotated regulatory element such as a non-coding RNA. Both *Camk1d* and *Plxdc2* are large genes, spanning more than the 400 kb on the rat genome. Indeed, there are three putative non-coding RNAs in the haplotype region encompassing *Camk1d* (*LOC102551026*, *LOC102551092*, and *LOC108348588*). In the rat rn6 annotation there are three non-coding RNAs in the haplotype region encompassing *Camk1d*; two of them have variants between LH and LN, but in each case the LH allele is the reference allele (ie the BN allele). Therefore we believe these are unlikely to be susceptibility factors for hypertension or hypertriglyceridemia. Interspersed in human *CAMK1D*, there exist three miRNAs and an uncharacterized non-coding (nc) RNA; there are also uncharacterized ncRNA transcripts in the mouse *Camk1d* gene region. However none of these non-coding RNAs are conserved in the rat. Still, important functional regulatory elements may be missed by focusing only on the annotated genes and transcripts in these regions. Furthermore, while causal variants are most likely due to selection of different ancestral DNA, it is also possible that the *de novo* variants arising since LH and LN diverged result in a phenotype. Future physiological and genome-editing studies will identify the causal variant(s) causing concurrent hypertension and dyslipidemia.

## Materials and methods

### Animals

Male animals used in this study were bred and maintained in an approved animal facility in micro-isolator caging on a 12-hour light-dark cycle at the University of Iowa. Unless otherwise noted, the rats were provided chow (Teklad 7913) and water *ad libidum*. Surgeries were performed under approved anesthesia (inhaled isoflurane or injected ketamine/xylazine) and euthanasia (CO_2_ inhalation) was performed using methodologies consistent with the recommendations of the American Veterinary Medical Association (AVMA) Guidelines for the Euthanasia of Animals. All animal protocols were reviewed and approved by the Institutional Animal Care and Use Committee (IACUC) at the University of Iowa.

#### Generation of consomic and congenic LH-17^LN^ rat strains

To generate the LH-17^LN^ consomic strain, LH × LN F1 animals were first generated by crossing LH/MRrrcAek male to LN/MRrrcAek female rats. LH × LN F1 males were then repeatedly backcrossed with LH females to fix the genome background. Male offspring from each backcross generation were genotyped with SNPs spanning RNO17 at 12–21 days of age, and only those animals with a fully heterozygous RNO17 were selected for subsequent generations. After the third backcross generation, offspring were genotyped with a panel of 453 SNPs spanning the genome that tagged all haplotypes that differ between the LH and LN strains. Males selected for the fourth through sixth generation were chosen based upon retaining a heterozygous RNO17 and enrichment for LH alleles in the genome background. Males and females heterozygous for SNPs spanning RNO17 and fixed for the LH genotype for all other regions of the genome were intercrossed to generate offspring where all SNPs on RNO17 were fixed for the LN allele, generating the consomic LH-Chr17^LN^/Aek (LH-17^LN^; RGD: 12903251) strain, as well as offspring with RNO17 recombinations from which the congenic strain LH.LH-Chr 17^LN^-(rs199194111-rs105876746)/Aek (LH-17^LN^c; RGD: 12903257) was derived. Offspring with desired recombination events on RNO17 were backcrossed to LH parental rats and then intercrossed to generate founder congenic strains. All rats used for phenotyping were genotyped to confirm their genotypes on RNO17 entering the phenotyping protocol. The regions of RNO17 substituted in the consomic and congenic rats are shown in Fig 1.

### SNP genotyping

DNA was isolated using DNEasy Blood and Tissue Kit (QIAGEN, Valencia, CA) according to manufacturer’s instructions. RNO17 SNP genotyping (S1 Table) was performed using custom TaqMan^®^ genotyping assays (Life Technologies), according to manufacturer’s instructions on StepOnePlus^®^ thermal cyclers (Applied Biosystems). For genome-wide genotyping during the generation of the consomic strain, a custom GoldenGate genotyping BeadChip array (Illumina, San Diego, CA) was used as previously described [19]. The array contains 1,536 SNPs, of which 453 SNPs are polymorphic between LH and LN at a resolution sufficient to determine the background genome was fixed for the LH allele. Genotyping was performed at GeneSeek (Neogen Corporation, Lincoln, NE). SNP calls with GenCall scores exceeding 0.4 were accepted for analysis.

### Phenotyping

Male LH-17^LN^ (N = 11), LH-17^LN^c (N = 6), and LH (N = 20) rats were characterized using a 15-week phenotyping protocol as previously described [19]. Briefly, starting at wean (21 days) male rats from each strain were weighed weekly. Two measures of length (nose to rump and nose to tip of tail) were also collected weekly for the first 8 weeks, then at 12, 16, and 18 weeks of age to provide an estimate of body weight relative to stature. At 12 weeks of age radiotelemeters (Data Sciences International) were implanted attached to a catheter in the femoral artery. At 14 weeks of age, heart rate (HR), systolic (SBP), diastolic (DBP) and mean arterial (MAP) blood pressure was measured for 72 consecutive hours (15 seconds every 10 minutes) in the animals’ home cage using the Dataquest ART 2.2 system (Data Sciences International). At 15 weeks of age the animals were switched to a 4% salt diet (TD.10141) and heart rate and blood pressure measures were repeated at 17 weeks of age. An average hourly measure was determined for each animal and used for statistical analysis. At 18 weeks of age, blood was collected before and after an overnight fast, the rats were humanely euthanized, and tissues including liver, kidney, skeletal muscle, fat, left ventricle of the heart, and hypothalamus were harvested and either snap frozen in liquid nitrogen or placed into RNAlater (Qiagen) for qPCR. Blood glucose was measured in unfasted and unanesthetized animals at 18 weeks of age using a hand held glucometer (Contour blood glucose meter, Bayer HealthCare LLC, Mishawaka, IN). Serum total, HDL and LDL cholesterol (BioAssay Systems) and triglyceride (BioVision, Inc) levels (mg/dL) were determined in fasted animals from the terminal blood draw. LH control rats were studied in parallel with the consomic and congenic cohorts. No phenotype differences were identified between the LH cohorts and their data was combined for analyses. Serum LDL levels in two consomic and one congenic rat was below the assay limit of detection of 1 mg/dL; for those samples the measure was set to 1 mg/dL. At the end of the study, data from two LH-17^LN^ rats were removed as additional genotyping identified a >10 Mb LH fragment at the distal end of the chromosome that was not previously tagged by available genetic markers.

For each phenotype, ROUT analysis was performed to identify outliers in the biological replicates with Q = 1% [42]. Differences between the LH, consomic, and congenic rats were determined using either a one-way (for single time point measures) or two-way ANOVA (for growth curve and hemodynamic measures) followed by post-hoc Holm-Sidak test to determine significant strain differences compared to the LH parental strain. GraphPad Prism^®^ software (GraphPad Software, Inc.) was used for all statistical analyses. Data is presented as mean ± SE.

### Quantitative real-time PCR (qPCR)

Tissues for studying gene expression were harvested at the end of the phenotyping protocol. The animals were 18 weeks of age and were on a 4% salt diet for 3 weeks. Total RNA was extracted from liver and kidney tissue from LH-17^LN^c (N = 11), LH-17^LN^ (N = 6), and LH (N = 8) rats, and cDNA was prepared (iScript cDNA synthesis kit, BioRad Life Sciences) for qPCR according to manufacturer recommendations. qPCR was performed for rat *Camk1d*, *Plxdc2*, and *β-Actin*, utilizing 5’ nuclease assays (PrimeTime Standard qPCR Assay, Integrated DNA Technologies, Inc., Coralville IA) on an ABI StepOnePlus (Applied Biosystems) and processed using Step One software (v2.3). All samples were run in technical triplicate and a mean CT calculated for each sample. Gene Expression was normalized using β-Actin as a housekeeping gene (ΔCt). Relative quantification (2^-ΔΔCt^) was calculated using the average ΔCt of the LH cohort. ROUT analysis was performed to identify outliers in the biological replicates with Q = 1% [42]. Gene expression differences between groups were determined by ANOVA followed by post-hoc Holm-Sidak tests for pairwise significance compared to the LH parental control.

## Supporting information

S1 Fig 24-hour hemodynamic measures in LH, LH-17^LN^ and LH-17^LN^c rats on chow (A-D) and 4% NaCl (E-H) diets. Dark cycle indicated by a gray box. LH: n = 20; LH-17^LN^: n = 9; LH-17^LN^c: n = 6. Data displayed as mean ± SE per hour across a 3-day measurement period. MAP = mean arterial blood pressure; SBP = systolic blood pressure; DBP = diastolic blood pressure; HR = heart rate.
(TIF)

S1 Table Assay information for custom TaqMan^®^ SNP genotyping. (XLSX)

S2 Table SNVs between LH and LN genomes in the LH-17^LN^ congenic region. (XLSX)
