# Supplementary figures and images for: Contribution of independent and pleiotropic genetic effects in the metabolic syndrome in a hypertensive rat
Source: PLoS One. 2017 Aug 8;12(8):e0182650. doi: 10.1371/journal.pone.0182650 (PMC5549746; doi:10.1371/journal.pone.0182650)

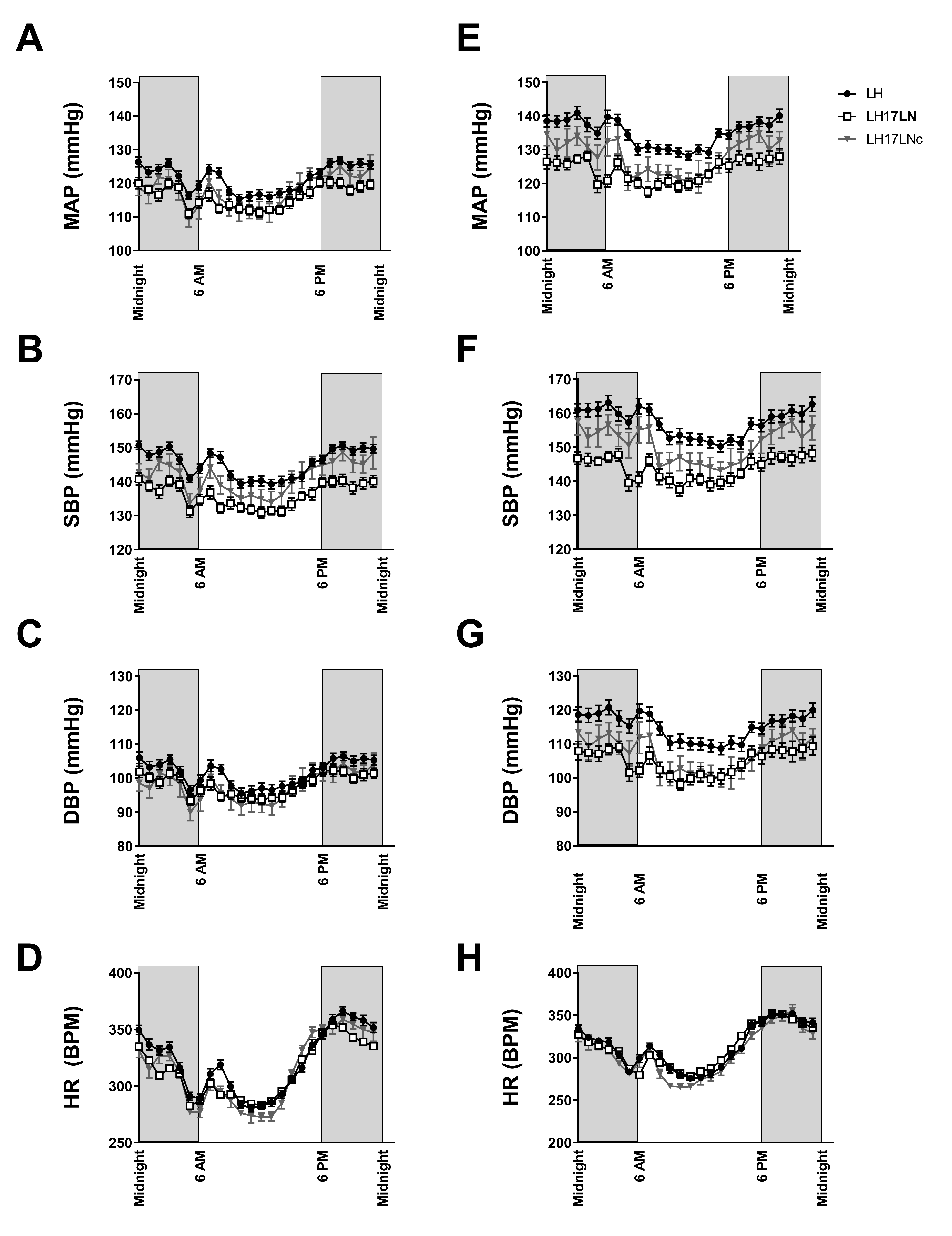

Supplement: S1 Fig — Dark cycle indicated by a gray box. LH: n = 20; LH-17LN: n = 9; LH-17LNc: n = 6. Data displayed as mean ± SE per hour across a 3-day measurement period. MAP = mean arterial blood pressure; SBP = systolic blood pressure; DBP = diastolic blood pressure; HR = heart rate. (TIF) [file pone.0182650.s001.tif]
